# A Novel m6A-Related LncRNA Signature for Predicting Prognosis, Chemotherapy and Immunotherapy Response in Patients with Lung Adenocarcinoma

**DOI:** 10.3390/cells11152399

**Published:** 2022-08-03

**Authors:** Yefeng Shen, Shaochun Wang, Yuanzhou Wu

**Affiliations:** 1 Institute for Pathology, University Hospital of Cologne, 50937 Cologne, Germany; shenyefeng2015@gmail.com; 2 Department of Thoracic Surgery, Zhujiang Hospital, Southern Medical University, Guangzhou 510282, China; 3 Department of Oncology, Shijiazhuang People’s Hospital, Shijiazhuang 050000, China; wsc91329@163.com

**Keywords:** lung adenocarcinoma, m6A, lncRNA score, immune checkpoints, immunotherapy

## Abstract

N6-methyladenosine (m6A) and long non-coding RNA (lncRNA) have been associated with cancer prognosis and the effect of immunotherapy. However, the roles of m6A-related lncRNAs in the prognosis and immunotherapy in lung adenocarcinoma (LUAD) patients remain unclear. We evaluated the m6A modification patterns of 695 samples based on m6A regulators, and prognostic m6A-related lncRNAs were identified via a weighted gene co-expression network analysis. Twelve abnormal m6A regulators and nine prognostic lncRNAs were identified. The tumor microenvironment cell-infiltrating characteristics of three m6A-related lncRNA clusters were highly consistent with the three immune phenotypes of tumors, including immune-excluded, immune-inflamed and immune-desert phenotypes. The lncRNA score system was established, and high lncRNA score patients were associated with better overall survival. The lncRNA score was correlated with the expression of the immune checkpoints. Two immunotherapy cohorts supported that the high lncRNA score enhanced the response to anti-PD-1/L1 immunotherapy and was remarkably correlated with the inflamed immune phenotype, showing significant therapeutic advantages and clinical benefits. Furthermore, the patients with high lncRNA scores were more sensitive to erlotinib and axitinib. The lncRNA score was associated with the expression of miRNA and the regulation of post-transcription. We constructed an applied lncRNA score-system to identify eligible LUAD patients for immunotherapy and predict the sensitivity to chemotherapeutic drugs.

## 1. Introduction

In 2021, lung cancer accounted for one-quarter of all of the cancer-related deaths on a global scale [1], and nearly 40% of all of the lung cancer cases fall into non-small cell lung cancer (NSCLC) [2]. Despite significant advances in cancer therapy, such as radiation therapy, chemotherapy, surgical resection and immunotherapy, which have made considerable progress in prolonging the survival of patients, the long-term prognosis for these patients remains unsatisfactory [3]. Therefore, it is essential to discover novel biomarkers and comprehensive insights into the mechanism for predicting an efficacious therapy for lung adenocarcinoma (LUAD). m6A, the methylation modification at the sixth position of the nitrogen atom of adenosine, is the most abundant modification of RNA. The m6A modification regulates the transcription, stability, splicing, degradation, localization, transport and translation of RNA [4,5]. The m6A modification is reversible and mediated by three types of regulators, including methyltransferases (writers), demethylases (erasers) and methylation recognition enzymes (readers). Therefore, m6A modification and regulators play vital roles in the carcinogenesis and the development of cancers, while novel mechanisms of the m6A modification remain largely unknown.

As crucial regulators in epigenetics, accumulating evidence has revealed that the long non-coding RNAs (lncRNAs) affect numerous biological processes with diverse mechanisms, including cell proliferation, metastatic progression [6], apoptosis [7] and the stemness and modulation of metabolism [8], especially in cancers. Moreover, the intracellular functions of the lncRNAs are mediated by the m6A regulators, indicating a complex and multiple interaction between the molecules. The lncRNA PRADX peroxiredoxin 1 (PRADX) promotes the nuclear factor-κB (NF-κB) activity via the UBX domain protein 1 (UBXN1) suppression, inducing the tumorigenesis of glioblastoma and colon adenocarcinoma by interacting with the enhancer of zeste homolog 2 (EZH2) [9]. Thus, the further identification of the m6A-related lncRNAs and an exploration of their functions in malignancies are imperative.

Immune checkpoint blockade (ICB) therapies, such as monoclonal antibodies against programmed death 1 (PD-1) or programmed death ligand 1 (PD-L1) and cytotoxic T-lymphocyte associated protein 4 (CTLA4), have achieved unprecedented efficacy in a wide range of malignancies through boosting the immune system to fight cancer. Notably, it has been shown that pembrolizumab is related to remarkably prolonged overall survival and a progression-free survival (PFS) duration in patients with advanced NSCLC patients, as well as PD-L1 expression on a minimum of 50% of tumor cells in contrast to platinum-based treatments [10]. Although the effect of treatment for lung cancer patients has been improved with the application of ICB-based immunotherapies, only a small proportion of individuals may gain benefit from immunotherapy. Hence, it is critical to predict and identify the best candidates for immunotherapy and provide individualized drug treatment.

Our study identified 12 differentially expressed m6A regulators, based on the expression between LUAD and the adjoining normal tissues. Nine hub m6A-related lncRNAs were detected from a key module by a weighted gene co-expression network analysis (WGCNA) and univariate Cox regression. We successfully identified three distinct m6A-related lncRNA subgroups, as well as three distinct lncRNA-related gene subtypes. The tumor microenvironment cell-infiltrating characteristics of the three m6A-related lncRNA clusters were highly consistent with the three immune phenotypes of the tumors. Moreover, the lncRNA score was constructed to predict the lncRNA modification in individuals and validated to anticipate the response to anti-PD-1/L1 immunotherapy and chemotherapeutic drugs. The lncRNA score was highly correlated with the expression of miRNA and the regulation of post-transcription. Therefore, our research established an applied scoring scheme, based on the m6A-related lncRNAs, to identify the LUAD patients who are eligible for immunotherapy and to predict sensitivity to chemotherapeutic drugs.

## 2. Materials and Methods

### 2.1. Data Acquisition

The Gene-Expression Omnibus (GEO) and the Cancer Genome Atlas (TCGA) databases were searched for the purpose of acquiring the LUAD RNA expression profile, along with the corresponding complete clinical annotations. A LUAD cohort, GSE43458 [11] containing 110 patients, was included for further analysis while two immunotherapy cohorts (IMvigor210 [12] and GSE78220 [13]) were also involved in our analysis. Table S1 (Supplementary Materials) provides a list of the cutoff thresholds that we used for the present research. The targeted mRNAs of the miRNAs were evaluated by FunRich 3.1.3. http://www.funrich.org/ (accessed on 25 February 2022) and the targeted signaling pathways of the miRNAs were enriched by the Kyoto Encyclopedia of Genes and Genomes (KEGG). Alternative polyadenylation (APA) was downloaded from the Cancer 3′ UTR Atlas (TC3A). http://tc3a.org (accessed on 28 March 2022) [14] and the alteration of the APA usage in each tumor can be quantified as a change in the distal poly(A) site-usage index (PDUI), identifying 3′UTR lengthening (positive index) or shortening (negative index) [15].

### 2.2. WGCNA

One thousand lncRNAs were chosen by median absolute deviation (MAD) to establish a co-expression network with the WGCNA package in R software to explore the relationship between the modules and m6A regulators. Following the deletion of the outliers at a cutoff threshold of 35 and with a minimum sample size of 50, the data were subjected to clustering with a hierarchical clustering algorithm. With the blockwise Modules function of the “WGCNA” package in R software, an unsigned network was created with the soft-threshold power adjusted to 5, the cut height adjusted to 0.1 and the minimum module size adjusted to 30 for the purposes of network formation and module detection.

### 2.3. Unsupervised Clustering for 9 LncRNAs and Principal Component Analysis (PCA)

The R package “limma” was utilized to standardize the data and identify lncRNAs with the prognostic values. The “ConsensusClusterPlus” package was used to conduct an unsupervised clustering algorithm on the lncRNAs for the purpose of classifying the LUAD patients into distinct subtypes based on the results of the study [16]. The number of clusters (K) and their stability were determined by the consensus clustering algorithm. The R package “PCA” was employed to verify the results of the grouping.

### 2.4. Gene Set Variation Analysis (GSVA)

To explore the different biological functions between the lncRNA subtypes, we conducted GSVA using the “GSVA” package in R software.

### 2.5. Identification of Differentially Expressed Genes (DEGs) between LncRNA Subtypes

To reveal the lncRNA-related genes, we classified patients into different gene subtypes based on the expression of genes. The empirical Bayesian approach of “limma” R package was applied to determine the DEGs between the different gene subgroups.

### 2.6. Establishment of LncRNA Score

We constructed a scoring system to quantitively determine the lncRNA-associated pattern in the individual LUAD patients and the lncRNA phenotype-related gene signature was named the lncRNA score. The genes with a prognostic significance were identified with the analysis of the Cox regression model. For the purpose of identifying the overlapping DEGs and classifying the patients into distinct subsets, an unsupervised clustering technique was utilized. The clusterProfiler R package was adopted to annotate the gene patterns. To define the number of clusters and their stability, the consensus clustering algorithm was applied. For the gene-expression analysis normalized by TPM methods, the expression of each gene in a signature was first transformed into a z-score. The lncRNA score was then constructed by separating the principal components (PC) 1 and 2 that were extracted to serve as the signature score. Subsequently, we computed each patient’s lncRNA score using a method similar to that which was used in the previous studies [17]:

(1) $$\mathrm{LncRNA score}\;=\;\sum ({PC1}_{i})\;+\;\sum ({PC2}_{i})$$

i indicates the expression of lncRNA-related genes. The patients were further classified into the low and high lncRNA score group according to the median score.

### 2.7. Mutation Profiles

The significantly mutated genes between the two low and high lncRNA score groups and the interaction effect of the gene mutations were analyzed with the maftools. The total number of nonsynonymous mutations in the TCGA-LUAD cohort was examined to determine the tumor mutation burden (TMB).

### 2.8. Prediction of Chemotherapeutic Drugs

To evaluate the different sensitivities to the chemotherapeutic agents for the high and low lncRNA score subgroups, the pRRophetic algorithm was conducted to predict the 50% inhibiting concentration (IC50) value of the 138 drugs, based on the Cancer Cell Line Encyclopedia (CCLE) [18].

### 2.9. Statistical Analysis

The concentrations of RNA in the tumor tissues and the adjoining normal tissues were compared by a Wilcox test. By performing the Kaplan–Meier analysis in conjunction with a log-rank test, we compared the OS of the various groups. The Cox regression of OS was conducted on the univariate data to discover the prognosis-related molecules. The R software (version: 4.0.5) was utilized for the purpose of conducting all of the analyses of statistical data and used for the generation of figures. All of the statistical tests were performed using a double-sided design, with *p* < 0.05 serving as the criterion for determining statistical significance.

## 3. Results

### 3.1. m6A-Related LncRNAs Associated with the Prognosis of LUAD

**Figure 1:**
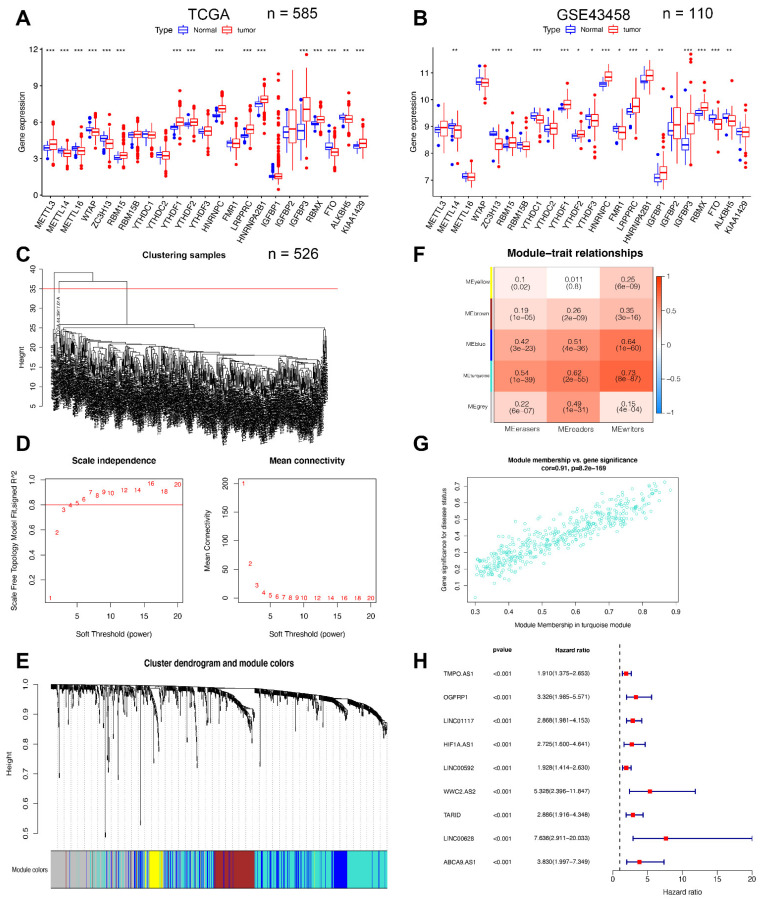
Identification of 9 lncRNAs associated with m6A regulators in the TCGA-LUAD cohort. Expression levels of m6A RNA methylation regulators between LUAD and adjacent normal tissues in (**A**) TCGA-LUAD (*n* = 585) and (**B**) GSE43458 (*n* = 110); (**C**) Clustering dendrogram of samples (*n* = 526) to detect outliers; (**D**) Scale independence and the mean connectivity of the WGCNA samples; (**E**) Dendrogram of all lncRNAs clustered with dissimilarity measure based on topological overlap; (**F**) Heatmap of the correlation between modules and m6A regulators. Each cell contains the correlation coefficient and *p*-value; (**G**) Scatter plot of lncRNAs in turquoise module; (**H**) Univariate Cox regression analysis for 9 lncRNAs from the turquoise module. * *p* < 0.05, ** *p* < 0.01, *** *p* < 0.001.

Figure S1 (Supplementary Materials) depicts the workflow of the present research. In the TCGA-LUAD cohort (Figure 1A) and the GSE43458 dataset (Figure 1B), the levels of METTL14, ZC3H13, FTO and ALKBH5 were consistently lower, while the levels of RBM15, YTHDF1, YTHDF2, HNRNPC, LRPPRC, HNRNPA2B1, IGF2BP3 and RBMX were higher in the tumor tissues as opposed to the adjacent tissues. Therefore, we selected 12 abnormally expressed m6A regulators for further detailed analysis.

Increasing evidence demonstrated that the lncRNAs play the key role in the progression of, and immunotherapy for, cancers [19], while the lncRNAs are regulated by the m6A regulators [20]. To elicit the correlation between the m6A regulators and lncRNAs, we performed the WGCNA on the TCGA-LUAD cohort, incorporating differentially expressed lncRNAs to identify the key module most closely related to the m6A regulators (Figure 1C). As shown in Figure 1D, beta (β) = 4 (scale-free R2 = 0.79, slope = −1.7) was set as the soft-threshold. A total of five modules were obtained after merging similar modules (Figure 1E). As shown in a heatmap of the module-trait relationships, the turquoise module containing 438 lncRNAs was considered as a novel module, and it was the most positively correlated with th em6A regulators including writers, erasers and readers (Figure 1F; Table S2, Supplementary Materials). Moreover, the turquoise module had the greatest module significance in all of the modules with the m6A writers (Figure S2A, Supplementary Materials), erasers (Figure S2B, Supplementary Materials) and readers (Figure S2C, Supplementary Materials), which indicated a strong correlation with the m6A modification. The correlation coefficient, as well as the *p*-value between the module membership and gene significance, were 0.91 and 8.2 × 10^−169^, respectively (Figure 1G). Hence, the turquoise module was the most positive module with the m6A regulators. To further determine the prognosis-related lncRNAs from the turquoise module, we performed a univariate Cox regression analysis and nine lncRNAs were detected (Figure 1H). High levels of the nine lncRNAs were significantly related to low OS rates in LUAD patients (Figure S2D, Supplementary Materials). Therefore, the nine m6A-related lncRNAs were identified with the prognosis of LUAD.

### 3.2. Three LncRNA Clusters Were Highly Consistent with the Three Immune Phenotypes

**Figure 2:**
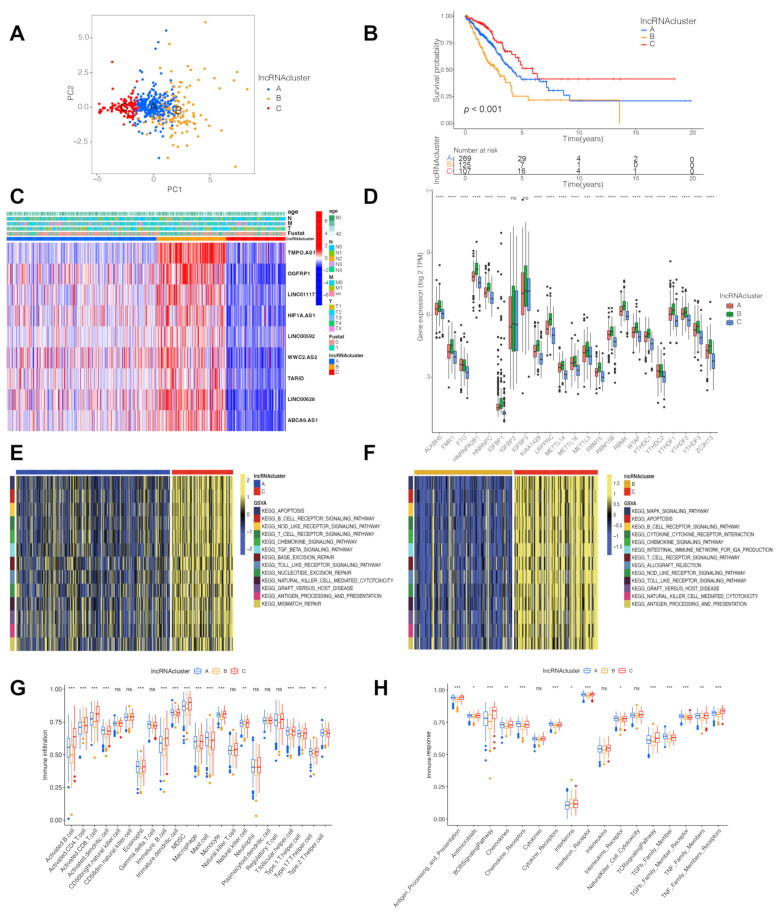
TME cell infiltration characteristics and transcriptome traits in distinct lncRNA clusters. (**A**) Remarkable difference between three lncRNA clusters was plotted via principal component analysis. Patients from TCGA-LUAD were divided into lncRNA cluster A/B/C. PC, principal component; (**B**) Kaplan–Meier curves of survival in TCGA-LUAD cohort with three lncRNA clusters; (**C**) Heatmap displaying clinical features and the expression distributions of 9 hub lncRNAs; (**D**) Expression levels of 23 m6A regulators in three lncRNA clusters, TPM, transcript per million; GSVA enrichment analysis showing the activation states of biological pathways in distinct lncRNA clusters. The heatmap was used to visualize these biological processes, and yellow represented activated pathways and blue represented inhibited pathways; (**E**) LncRNA cluster A vs C; (**F**) LncRNA cluster B vs C; (**G**) Characteristics of immune-infiltrating cells in different lncRNA clusters; (**H**) Characteristics of immune responses in different lncRNA clusters. *** *P* < 0.001, **** *P* < 0.0001, ns, not significant.

By conducting unsupervised clustering according to the levels of the nine lncRNAs, the patients from the TCGA-LUAD were divided into three subtypes, named lncRNA clusters A/B/C (Figure S3A–C, Supplementary Materials). The PCA results determined that a relatively evident distinction existed in the three clusters (Figure 2A). A better prognosis was indicated for the lncRNA cluster C than for the lncRNA clusters A/B (Figure 2B). In addition, the heatmap indicated the clinicopathological implications and the levels of the nine lncRNAs (Figure 2C), while the expression of the 23 m6A regulators differed remarkably in the three clusters (Figure 2D).

To identify the biological roles of the three lncRNA clusters, GSVA enrichment pathways were conducted. Compared with the lncRNA clusters A and B, the lncRNA cluster C was associated with immune full activation including the B and T cell receptor-signaling pathway, the chemokine signaling pathway, the natural killer cell-mediated cytotoxicity and the Toll-like receptor signaling pathway (Figure 2E,F). In addition, the lncRNA cluster C was rich in the infiltration of the various activated immune cells (Figure 2G). Considering a matching survival advantage, the lncRNA cluster C was classified as an immune-inflamed phenotype, characterized by adaptive immune cell infiltration and immune activation. Even though the lncRNA cluster A was correlated with the immune suppression process (Figure 2E), lncRNA cluster A was relatively highly correlated with the innate immune cells, including macrophage, mast cell, monocyte, natural killer, eosinophil and MDSC (Figure 2G). Strikingly, the lncRNA cluster A was extremely associated with the TGF-β family member and TGF-β family member receptor (Figure 2H). Numerous studies revealed that the immune-excluded phenotype was characterized by the presence of abundant immune cells and the upregulation of the TGF-β signaling pathway, while the immune cells were hindered in the stroma surrounding the nests of tumor cells and do not penetrate the parenchyma of the tumors [21,22]. Interestingly, the lncRNA cluster A was considered as the immune-excluded phenotype. Furthermore, the lncRNA cluster B was determined with few immune cells and the suppression of the immune response (Figure 2F–H), in accordance with the main characteristics of the immune-desert phenotype. Therefore, the three lncRNA clusters presented a significantly distinct tumor microenvironment (TME) cell-infiltration characterization.

### 3.3. Identification of LncRNA-Related Gene Subtypes and Construction of LncRNA Score

**Figure 3:**
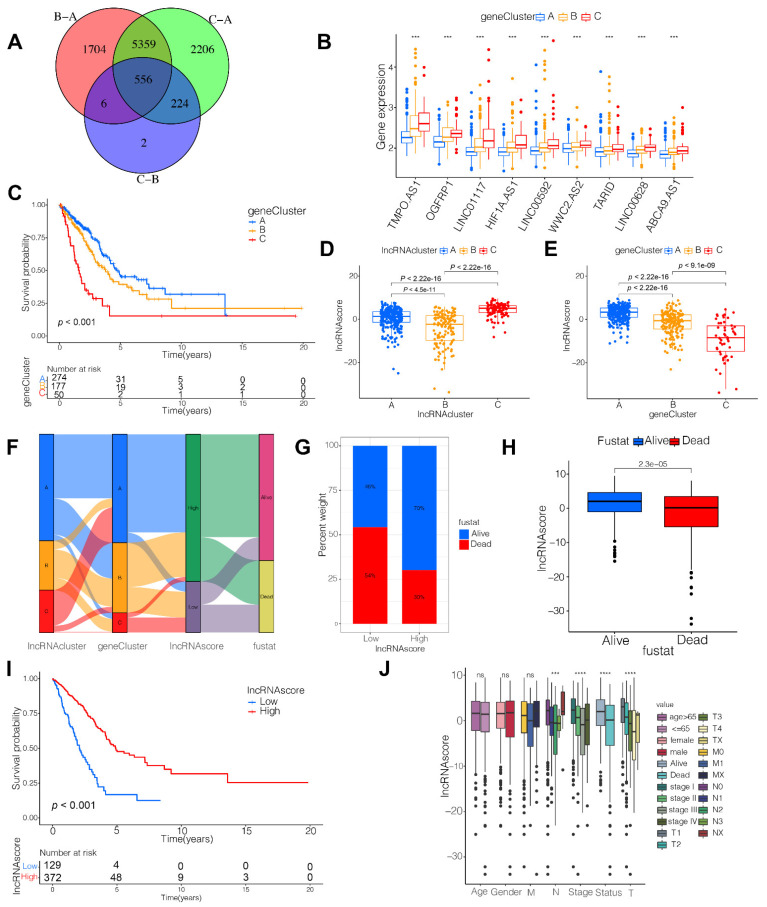
Construction of lncRNA score and the prognostic values of lncRNA score. (**A**) A total of 556 lncRNA cluster-related genes shown in Venn diagram; (**B**) Levels of 9 hub lncRNAs in the three gene clusters; (**C**) Kaplan–Meier curves of survival in the TCGA-LUAD (*n* = 501) cohort with three distinct gene clusters; LncRNA score in distinct (**D**) lncRNA clusters and (**E**) gene clusters; (**F**) Alluvial diagram showing the changes in lncRNA clusters, gene clusters and lncRNA scores; (**G**) Proportion of survival and death in the high and low lncRNA score groups; (**H**) Comparison of the lncRNA score in alive versus dead patients; (**I**) Kaplan–Meier curves of survival in the high and low lncRNA score groups; (**J**) Difference in lncRNA score among distinct clinical subgroups in LUAD cohort. *** *p* < 0.001, **** *p* < 0.0001, ns, not significant.

To investigate the potential genetic changes based on the distinct lncRNA subsets, we obtained 556 overlapped DEGs (Figure 3A) and identified 105 DEGs with the prognostic values by a univariate Cox regression analysis (Table S3, Supplementary Materials). We then performed an unsupervised cluster analysis and divided the patients into three distinct genomic subtypes, defined as gene clusters A/B/C (Figure 3D–F and Figure S3, Supplementary Materials). The levels of the nine hub lncRNAs differed significantly in the three gene clusters (Figure 3B), while the clinicopathological characteristics of those gene clusters are shown in Figure S3G (Supplementary Materials). Strikingly, there was a dramatically improved prognosis in gene cluster A than in the other clusters (Figure 3C).

Although our data revealed lncRNA-related gene modification in the prognosis, we considered constructing applied scores to predict the lncRNA modification in individuals, based on the 105 lncRNA-related DEGs. We found on the evaluation that the patients in the lncRNA cluster C (Figure 3D) and gene cluster A (Figure 3E) had high lncRNA scores. The process of constructing the lncRNA scores is depicted in an alluvial diagram (Figure 3F). Furthermore, we explored an overlap analysis of the three subtypes. There were 51.8% of the patients in the high lncRNA score group overlapped with the lncRNA cluster A, and 38.4% of the samples in the low lncRNA score group overlapped with the lncRNA cluster B (Figure S4A, Supplementary Materials). Meanwhile, seventy-six percent of the cases in the high lncRNA score group overlapped with the gene cluster A, while 48.8% of the patients in the low lncRNA score group overlapped with gene cluster B (Figure S4B, Supplementary Materials). The survival rate in the high lncRNA score group was much higher as opposed to that in the low lncRNA score group (70% vs 46%; Figure 3G), similar with the results at early- (T1-2) and advanced- (T3-4) stage lung cancer (Figure S4C,D, Supplementary Materials). Consistent with this finding, the average lncRNA scores were significantly higher in the live cases than those in the dead cases (Figure 3H). The results from the Kaplan–Meier analysis indicated a favorable prognosis for patients in the high lncRNA score group (Figure 3I; *P* < 0.001). Moreover, it was determined that the patients with high lncRNA scores were correlated with early clinicopathological features and stages (Figure 3J), which suggested that these patients were characterized by the lncRNA cluster C and the immune-inflamed phenotype with a survival advantage. Considering the univariable and multivariate Cox regression analyses, the lncRNA score independently served as a prognostic indicator (Figure S4E,F, Supplementary Materials). The nomogram shows that the lncRNA score was a predicted biomarker for LUAD (Figure S4G, Supplementary Materials).

### 3.4. LncRNA Score Associated with Immune Checkpoints

**Figure 4:**
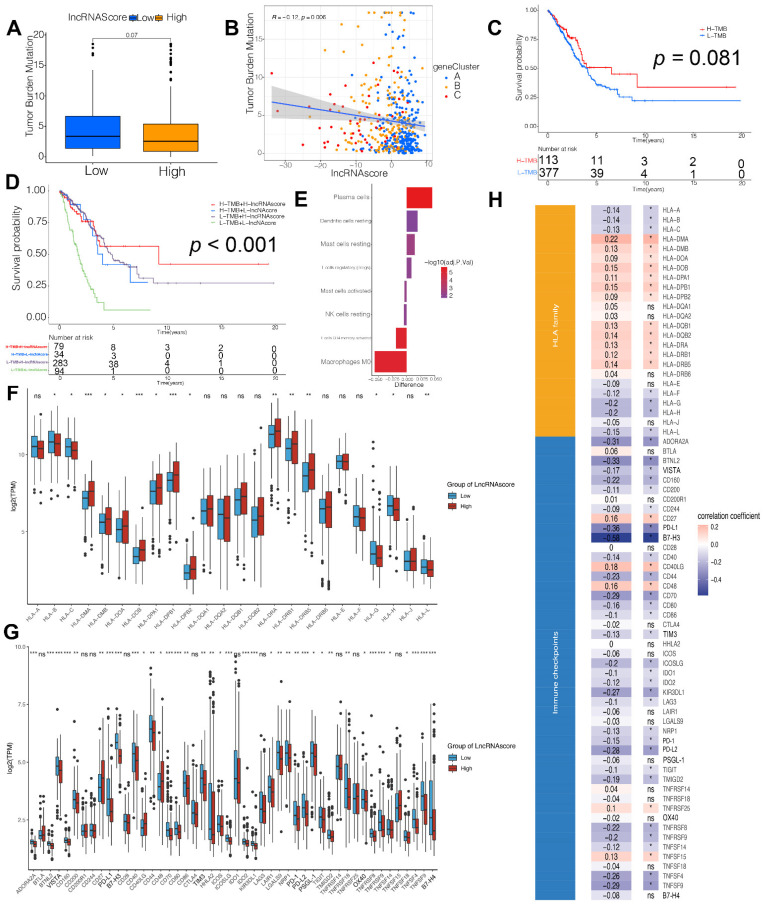
LncRNA score correlated with immune checkpoints. (**A**) Comparison of tumor mutation burden (TMB) in the high and low lncRNA score group; (**B**) Correlation between the lncRNA score and TMB; (**C**) Kaplan–Meier curves of survival in the high and low TMB groups; (**D**) Survival analyses for patients stratified by both lncRNA score and TMB using Kaplan–Meier curves; (**E**) Difference in the relative abundance of immune cell infiltration in TME between the high and low lncRNA score groups. Difference > 0 indicates that the immune cells were enriched in the low lncRNA score group, and the column color represents the statistical significance of the difference.; Analyses for (**F**) the expression of HLA family genes and (**G**) immune checkpoints in the lncRNA score groups; (**H**) Correlation analysis for lncRNA score and the expression of HLA family genes/immune checkpoints. * *p* < 0.05, ** *p* < 0.01, *** *p* < 0.001; ns, not significant; TPM, transcript per million.

To examine the possible mechanisms of the lncRNA score in LUAD, immunotherapy-related factors, including TMB and immune checkpoints, were analyzed in our study. Even though TMB did not modulate in the low and high lncRNA score groups (Figure 4A), the lncRNA score was also positively correlated with TMB (Figure 4B). There were no differences between the high and low TMB subgroups (Figure 4C). However, considering the combination of the TMB and lncRNA scores, we found that both a high lncRNA score and high TMB patients exhibited a favorable prognosis, in contrast with those in the low lncRNA score group (Figure 4D). As shown in Figure S4H (Supplementary Materials), the lncRNA score was associated with tumor-infiltrating immune cell types, including activated B cells, activated CD4 T cells and monocytes (Figure S4H, Supplementary Materials). The difference in the TME cells between the two lncRNA score groups was also explored. It was found that the infiltration by the plasma cells, resting dendric cells, resting mast cells and regulator T cells was higher in the low lncRNA score group, while the activated mast cells, activated CD4 T cells and macrophages were highly enriched in the high lncRNA score group (Figure 4E), indicating that the patients with the high lncRNA scores were immune activated. Our data provided the evidence that the lncRNA score was related to the immune signature, including TMB and infiltrating immune cells.

According to the Wilcoxon test, the 15 HLA family genes (Figure 4F) and 38 immune checkpoints (Figure 4G) varied significantly between the two lncRNA score groups. Moreover, the lncRNA score was strongly associated with 19 HLA family genes and 34 immune checkpoint expression levels (Figure 4H). In summary, these results indicated that the lncRNA score was strongly correlated with the tumor immune checkpoints.

### 3.5. LncRNA Score Predicted Immunotherapeutic Benefits

**Figure 5:**
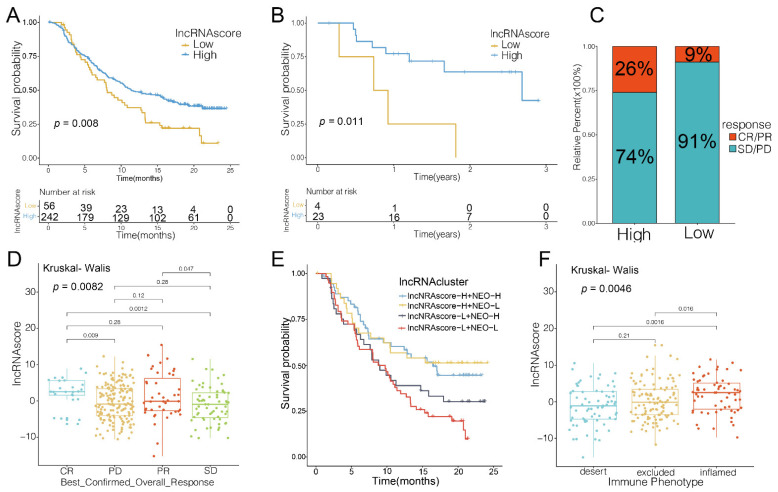
LncRNA score in the role of anti-PD-1/L1 immunotherapy. Kaplan–Meier curves of survival in the high and low lncRNA groups in the patients receiving (**A**) anti-PD-L1 therapy and (**B**) anti-PD-1 therapy; (**C**) Proportion of patients with response to PD-L1 blockade immunotherapy in the high and low lncRNA score groups; (**D**) Distribution of lncRNA score in distinct anti-PD-L1 clinical response groups; (**E**) Survival analyses for patients receiving anti-PD-L1 immunotherapy stratified by the combination of lncRNA score and neoantigen burden, using Kaplan–Meier curves; (**F**) Differences in lncRNA score among distinct tumor immune phenotypes in IMvigor210 cohort. SD, stable disease; PD, progressive disease; CR, complete response; PR, partial response; NEO, neoantigen burden.

We explored the predictive significance of the lncRNA scores for the responsiveness to ICB treatment in two immunotherapy groups. The patients with the high lncRNA scores exhibited a more favorable prognosis condition in contrast to those in the low lncRNA score group with anti-PD-L1 (IMvigor210, Figure 5A) and anti-PD-1 (GSE78220, Figure 5B) treatment. The patients with the high lncRNA scores had remarkable therapeutic benefits and enhanced immune responsiveness to the PD-L1 blockade (Figure 5C,D). Furthermore, it was shown that the patients who had a combined high lncRNA score and low neoantigen load benefited significantly in terms of survival (Figure 5E). In IMvigor210, the high lncRNA scores were significantly associated with the inflamed immune phenotype, and the checkpoint inhibitors exerted an antitumor effect in this phenotype (Figure 5F). Therefore, the lncRNA score was shown to be significantly correlated with the tumor immune phenotypes and useful in predicting the response to anti-PD1/L1 immunotherapy.

### 3.6. Mutation Status in the High and Low LncRNA Score Groups

**Figure 6:**
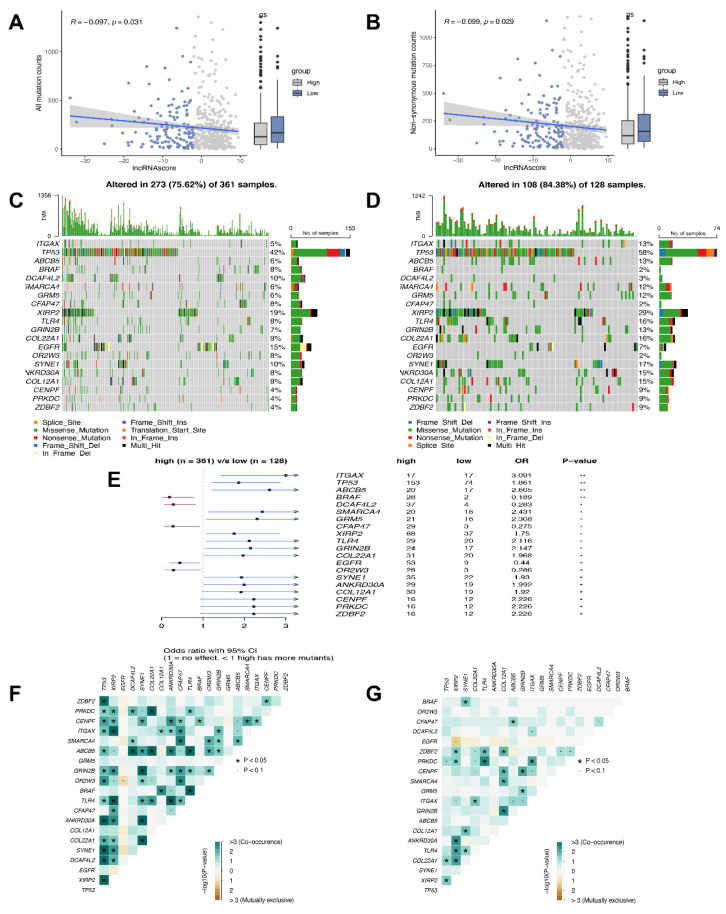
LncRNA score was related to tumor mutation status. Association between (**A**) all mutation counts, (**B**) non-synonymous mutation counts and the lncRNA score in the high and low lncRNA score groups; Visual summary showing common genetic alterations in the (**C**) high and (**D**) low lncRNA score groups; (**E**) Forest plot of gene mutations in the patients; Interaction effect of genes mutating differentially in patients in the (**F**) high and (**G**) low lncRNA score groups. * *p* < 0.05, ** *p* < 0.01.

To further determine the lncRNA score-related mechanisms in LUAD, more of the somatic mutations and non-synonymous mutations were identified in the low lncRNA score group (Figure 6A,B). The frequently mutated genes are shown in Figure 6C,D. Notably, five genes (*BRAF, DCAF4L2, CFAP47, EGFR* and *OR2W3*) mutated more frequently in the patients with high lncRNA scores. Fifteen genes were frequently mutated in patients in the low lncRNA score group, including *ITGAX, TP53, ABCB5, SMARCA4, GRM5, XIRP2, TLR4, GRIN2B, COL22A1, SYNE1, ANKRD30A, COL12A1, CENPF, PRKDC* and *ZDBF2* (Figure 6E). In addition, significant co-occurrences were found among the mutations of these genes in the high (Figure 6F) and low lncRNA score subgroups (Figure 6G). 

### 3.7. LncRNA Score Predicted the Sensitivity to Chemotherapeutic Drugs

**Figure 7:**
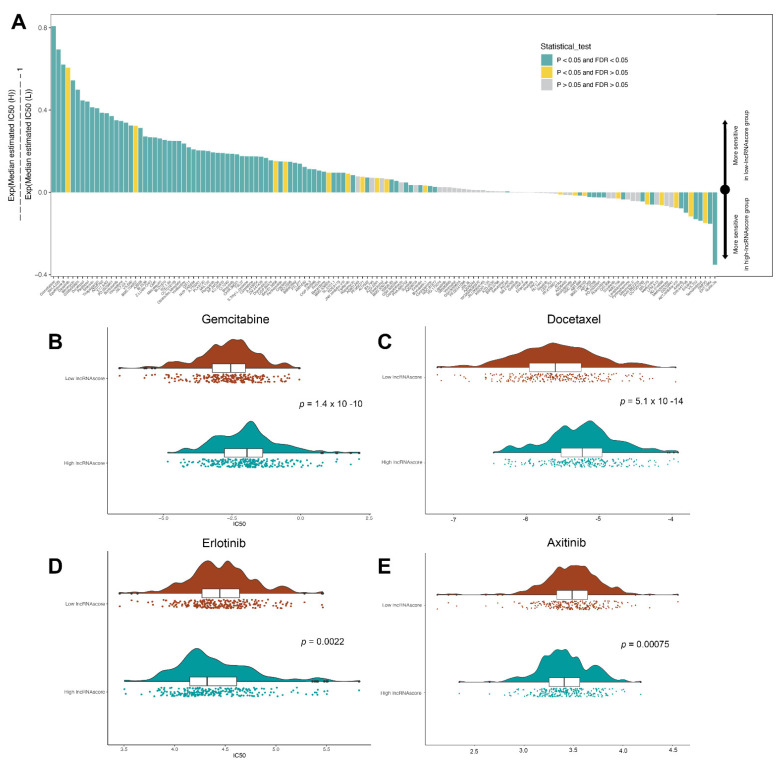
LncRNA score in the prediction of drug therapies. (**A**) Sensitivity of 138 drugs; the efficacy of (**B**) Gemcitabine; (**C**) Docetaxel; (**D**) Erlotinib and (**E**) Axitinib for LUAD patients. IC50, half maximal inhibitory concentration.

To evaluate the value of the lncRNA score for predicting the response to drugs, the IC50 values of 138 drugs were calculated (Figure 7A; Table S4, Supplementary Materials). We found that the low lncRNA score patients had a greater sensitivity to gemcitabine (Figure 7B), docetaxel (Figure 7C), cisplatin and paclitaxel, while those in the high lncRNA score group exhibited a greater sensitivity to erlotinib (Figure 7D) and axitinib (Figure 7E), suggesting that the lncRNA score was a predictive biological marker for medications against LUAD.

### 3.8. LncRNA Score Was Correlated with MiRNA and Post-Transcriptional Regulation

**Figure 8:**
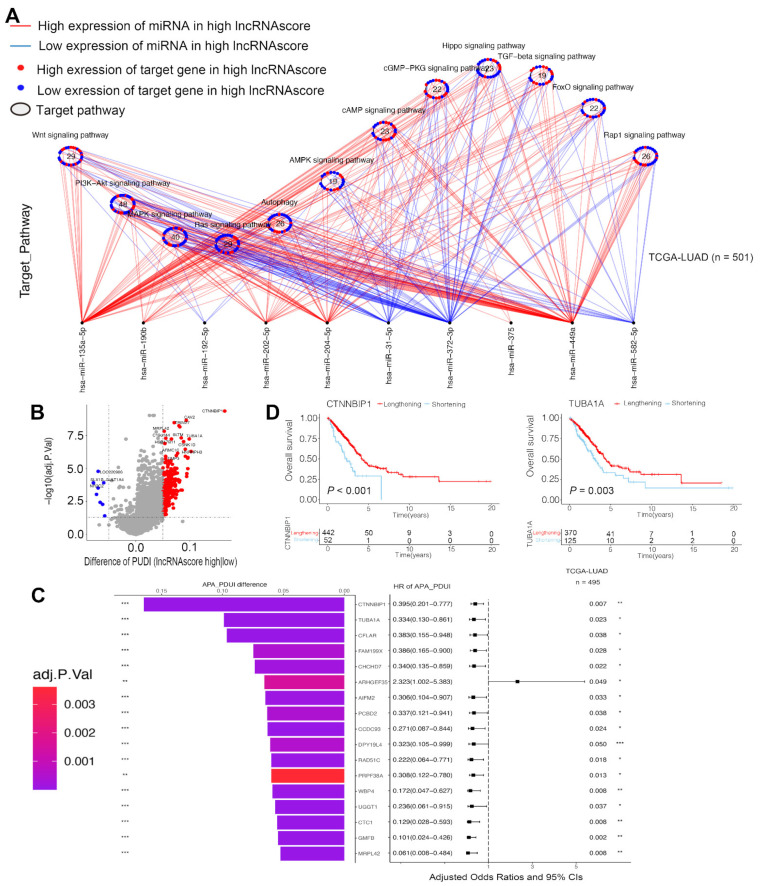
LncRNA score associated with the post-transcriptional characteristics. (**A**) Differences in miRNA-targeted signaling pathways in the TCGA-LUAD cohort between the high and low lncRNA score groups; (**B**) Differences in PDUI of each gene between the high and low lncRNA score groups; (**C**) The bar graphs showed the difference in the distal poly(A) site usage index (PDUI), and the forest plots showed univariate Cox regression analyses for PDUI differential genes between the high and low lncRNA score groups; (**D**) Kaplan–Meier curves indicated overall survival between PDUI lengthening (red) and PDUI shortening (blue) of CTNNBIP1 and TUBA1A. * *p* < 0.05, ** *p* < 0.01, *** *p* < 0.001.

It has been found that the m6A peaks are enriched at the miRNA target sites and the m6A RNA methylation is regulated by the miRNAs, so we hypothesized that the lncRNA score strongly associates with the expression of miRNAs as potential mechanisms. In the TCGA-LUAD cohort, we identified 33 differentially expressed miRNAs between the high and low lncRNA score groups. The miRNA-targeted genes were enriched in the PI3K-Akt signaling pathway, autophagy and other pathways (Figure 8A). Seven out of twenty-six miRNA-targeted genes in autophagy were highly expressed, while the targeted genes of the miRNAs with a lower expression in the high lncRNA score group were enriched in the cAMP signaling pathway (11/23) and cGMP-PKG signaling pathway (11/22). Our data indicated that the lncRNA score was significantly correlated with the miRNA expression and the regulation of the signaling pathways.

To explore the association between the lncRNA score and the post-transcription characteristics, we analyzed the APA events in the TCGA-LUAD. We identified the genes with the differences in APA between the high and low lncRNA score groups and explored the prognostic values to reveal whether the length of 3′UTR affects the survival of LUAD patients (Figure 8B). The genes with lengthening APA events were in the low lncRNA score group, corresponding to poor survival (Figure 8C). CTNNBIP1 [23] and TUBA1A [24] were considered as proto-oncogenes in some of the cancers and the short transcript of two genes was related to the poor survival of the LUAD patients (Figure 8D). Moreover, CTNNBIP1 was targeted directly by miR-29b on 3′UTR [25]. We held the belief that, due to the 3′UTR shortening of the genes, the miRNA might not be able to bind to the genes, relieving the inhibition to proto-oncogenes and leading to the development of LUAD.

## 4. Discussion

In the present research, we discovered 12 m6A regulators that were expressed differently between the LUAD and the adjacent normal tissues from the TCGA and GEO datasets. Considering the vital roles of the lncRNAs in the tumorigenesis and progression in cancers [26] and their mediation by the m6A regulator [27,28], we conducted a WGCNA to identify the m6A-related lncRNA module. A turquoise module was detected as a key module that is strongly related to the m6A regulators and nine hub lncRNAs were identified through a univariate Cox regression analysis. These nine lncRNAs were remarkably correlated with the OS of the LUAD patients, which aligns with the findings in other studies [29]. We then determined three distinct m6A modification-related lncRNA clusters. Three of the lncRNA clusters presented significantly different TME cell-infiltration characterization. The lncRNA cluster C correlated with immune activation and favorable prognosis, considered as an immune-inflamed phenotype. The lncRNA cluster A was characterized by the presence of abundant innate immune cells and the activation of the TGF-β signaling pathway, corresponding to an immune-excluded phenotype. The immune cells do not penetrate the parenchyma of these tumors but instead are retained in the stroma that surrounds the nests of tumor cells [30], leading to no improvement in survival. The lncRNA cluster B was immune suppressed, corresponding to an immune-desert phenotype. Hence, the TME cell-infiltrating characteristics under the three lncRNA clusters were strongly consistent with three immune phenotypes.

To explore the potential genetic changes based on the distinct lncRNA clusters, the patients were divided into three gene clusters. With consideration of the heterogeneity and complexity of the individuals, an applied and reliable scoring system, the lncRNA score, was constructed and used in the quantification of the lncRNA-associated pattern of each patient, based on the expression of DEGs. Notably, the patients with a high lncRNA score were found to have a favorable prognosis. The Cox regression analysis for both the univariate and multivariate models indicated that the lncRNA score independently acted as a prognostic indicator for the LUAD patients. Moreover, the remarkably prolonged survival of the group with a high lncRNA score and high TMB highlighted the benefit of a high lncRNA score. It is well known that TMB and the expression of immune checkpoints affect the efficacy of immunotherapy [31]. It was noted that some roles of the HLA family and vital genes, including PD-1, PD-L1, and TIM3 and B7-H4, were expressed differently in the high and low lncRNA score groups. Moreover, a remarkable correlation was found between the immune checkpoints and the lncRNA score. Thus, all of the above data revealed that the lncRNA score was involved in immunotherapy for the LUAD patients.

Immunotherapy is an emerging novel treatment for several cancers, especially lung adenocarcinoma. To validate our hypothesis that the lncRNA score is a reliable scoring system to identify the LUAD patients eligible for immunotherapy, we applied the lncRNA score in two immunotherapy cohorts. A high lncRNA score was correlated with a favorable patient prognosis in the anti-PD-L1 (IMvigor210) and anti-PD-1 (GSE78220) treatment groups. The PD-L1 blockade proved to have better therapeutic advantages and immune responses in the patients with a high lncRNA score. Furthermore, the combination of a high m6A score and a low neo-antigen burden served as a significant predictor of survival. Strikingly, higher lncRNA scores were dramatically associated with an inflamed immune phenotype, which provided the evidence that the high lncRNA score were responsible for immunotherapy. A combination of the results from the two immunotherapy cohorts highly supported the supposition that the lncRNA score is a predictor of the immunotherapeutic response in the LUAD patients. 

Nevertheless, mutation is an inescapable factor of the treatment effect from immunotherapy [32]. The patients with low lncRNA scores had a worse prognosis and carried more mutations in TP53, ITGAX and ABCB5. Some of the studies have shown that the TP53 mutations often inhibited antitumor immunity and the response to cancer immunotherapy [33,34,35], which aligns with our findings. Furthermore, the PD-1 inhibitors demonstrated profound clinical advantages when used in conjunction with the co-occurring mutations [36]. Fewer co-mutations occurred in the low lncRNA score group with the poor effect of immunotherapy, consistent with our previous results. Considering the traditional first-line treatment, the lncRNA score is a useful tool to predict the effect of chemotherapy. The low lncRNA score patients were more sensitive to gemcitabine, paclitaxel, docetaxel and cisplatin, whereas those with high lncRNA scores were more sensitive to erlotinib and axitinib. Overall, the lncRNA score was shown to serve as a predictor of clinical responsiveness to immunotherapy and a meaningful tool to evaluate drug sensitivity for the LUAD patients. To explore the possible mechanism of the lncRNA score, we found the lncRNA score, associated with the expression of miRNA and miRNA, might target the 3′UTR of genes, regulating the levels of the genes and contributing to the progress of the cancers.

## 5. Conclusions

In summary, we found an abnormal expression of 23 m6A RNA regulators in the LUAD and adjacent normal tissues. Three LUAD subtypes were obtained through the consensual clustering of m6A-mediated lncRNA, and three gene clusters were classified based on the lncRNA-related DEGs. We constructed a lncRNA score model to predict the prognosis of LUAD patients, which was highly associated with immune checkpoints and mutations. Notably, the lncRNA score was an applied score system to identify LUAD eligible patients for immunotherapy and predict sensitivity to chemotherapeutic drugs.

## Data Availability

The datasets analyzed for this study can be found in the TCGA-LUAD (http://www.cancer.gov/tcga, accessed on 25 February 2022) and GSE43458 (https://ftp.ncbi.nlm.nih.gov/geo/series/GSE43nnn/GSE43458/matrix/, accessed on 25 February 2022), IMvigor210 (https://clinicaltrials.gov/ct2/show/NCT02108652, accessed on 25 February 2022) and GSE78220 (https://ftp.ncbi.nlm.nih.gov/geo/series/GSE78nnn/GSE78220/matrix/, accessed on 25 February 2022).

## Supplementary Materials

The following supporting information can be downloaded at: https://www.mdpi.com/article/10.3390/cells11152399/s1, Table S1: The cut-off values used in the study; Table S2: Expression of differentially expressed lncRNAs in the patients in the turquoise module; Table S3: LncRNA-related DEGs in the patients; Table S4; Sensitivity of 138 drugs for LUAD patients. Kruskal–Wallis test was applied to analyze the *p*-value; Figure S1: Overview of our study; Figure S2: Nine lncRNAs were highly correlated with the survival of LUAD patients; Figure S3: Unsupervised clustering of lncRNAs and DEGs; (A) Consensus matrices of TCGA-LUAD cohort for k = 2 to 5; Figure S4: Prognostic value of lncRNA score and correlation between the clinicopathological features and lncRNA score.

## Abbreviations

LUAD: lung adenocarcinoma; NSCLC: non-small cell lung cancer; lncRNA: long non-coding RNA; ICB: immune checkpoint blockade; PD-1: programmed death 1; PD-L1: programmed death ligand 1; TCGA: The Cancer Genome Atlas; WGCNA: weighted gene co-expression network analysis; DEG: differentially expressed genes; TME: tumor microenvironment; TMB: tumor mutation burden.
