# Supplementary material for: A Novel m6A-Related LncRNA Signature for Predicting Prognosis, Chemotherapy and Immunotherapy Response in Patients with Lung Adenocarcinoma
Source: Cells. 2022 Aug 3;11(15):2399. doi: 10.3390/cells11152399 (PMC9368324; doi:10.3390/cells11152399)
Supplement: Supplementary file 1 [file cells-11-02399-s001.zip › cells-1809121-supplementary-figures.pdf]

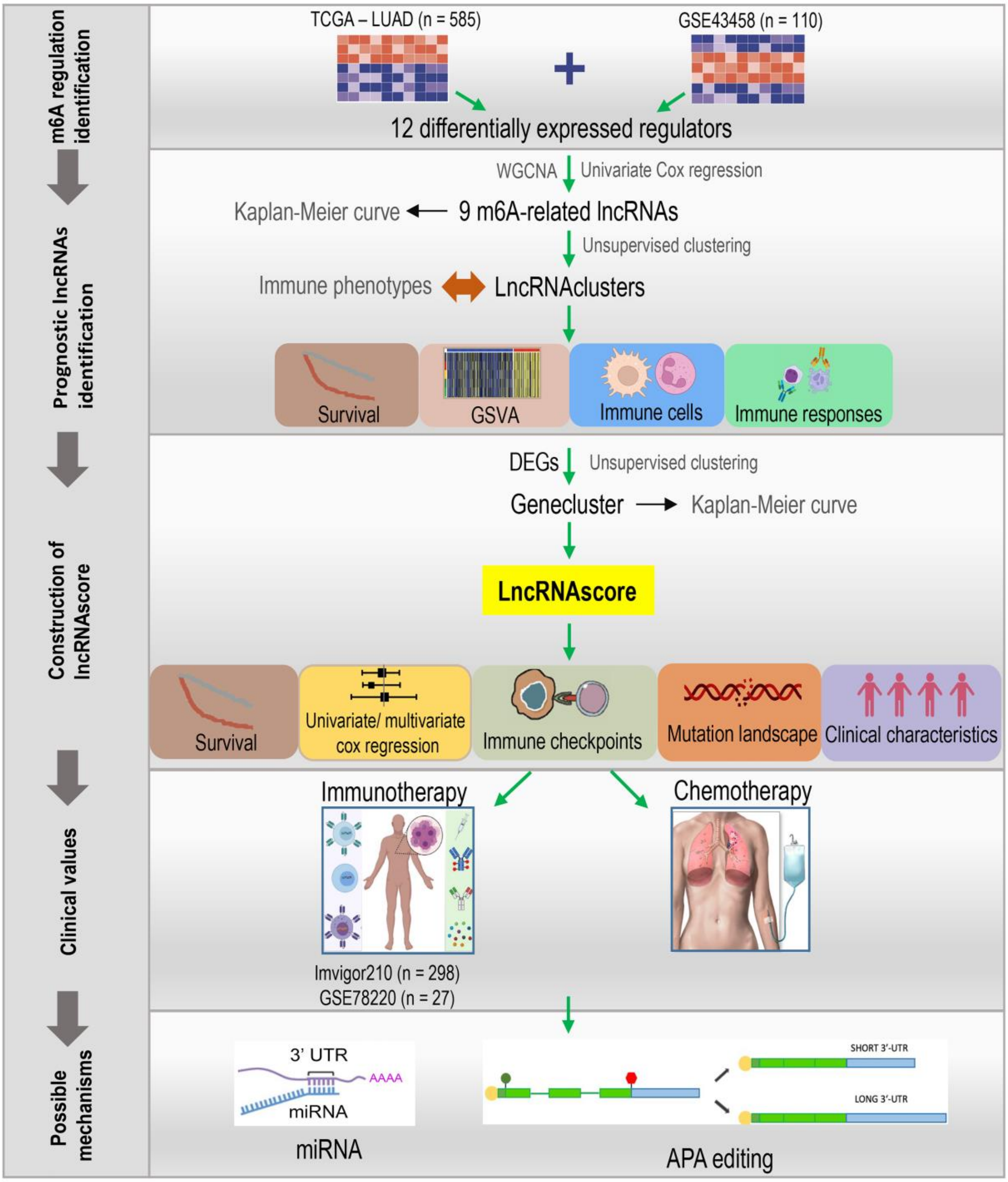

Figure S1. Overview of our study.

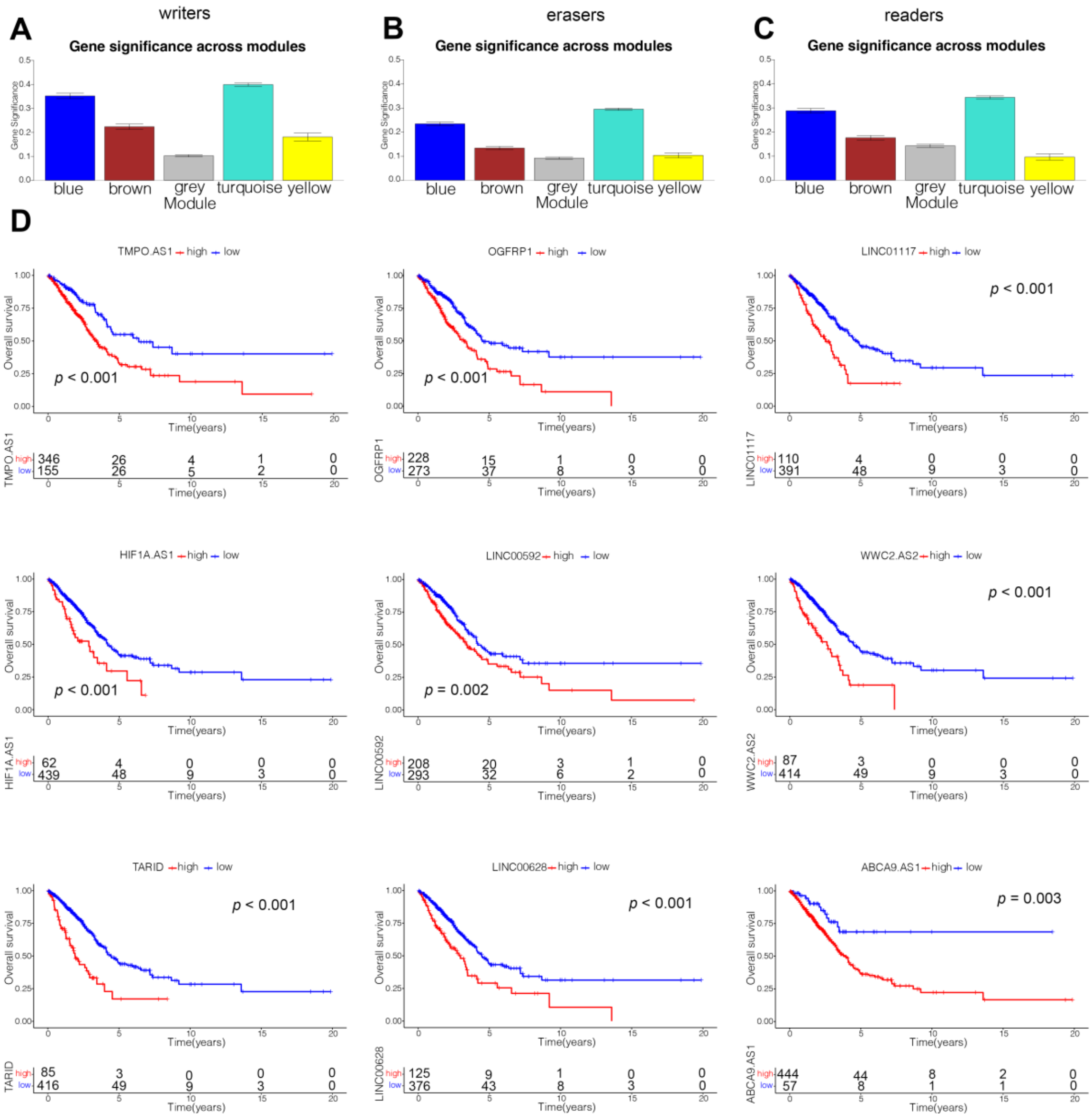

Figure S2. Nine lncRNAs were highly correlated with the survival of LUAD patients.

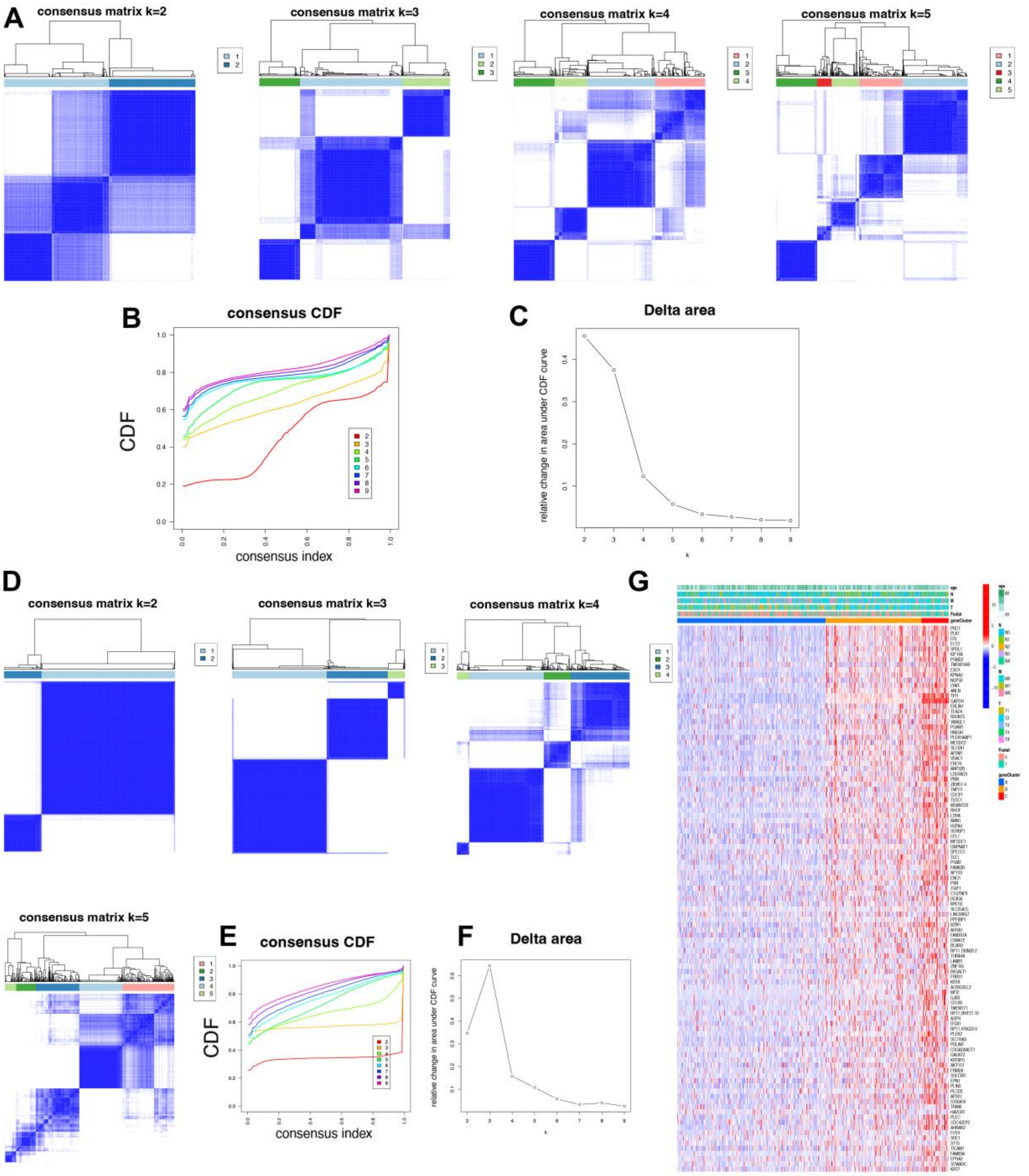

Figure S3. Unsupervised clustering of lncRNAs and DEGs; (A) Consensus matrices of TCGA-LUAD cohort for k = 2 to 5.

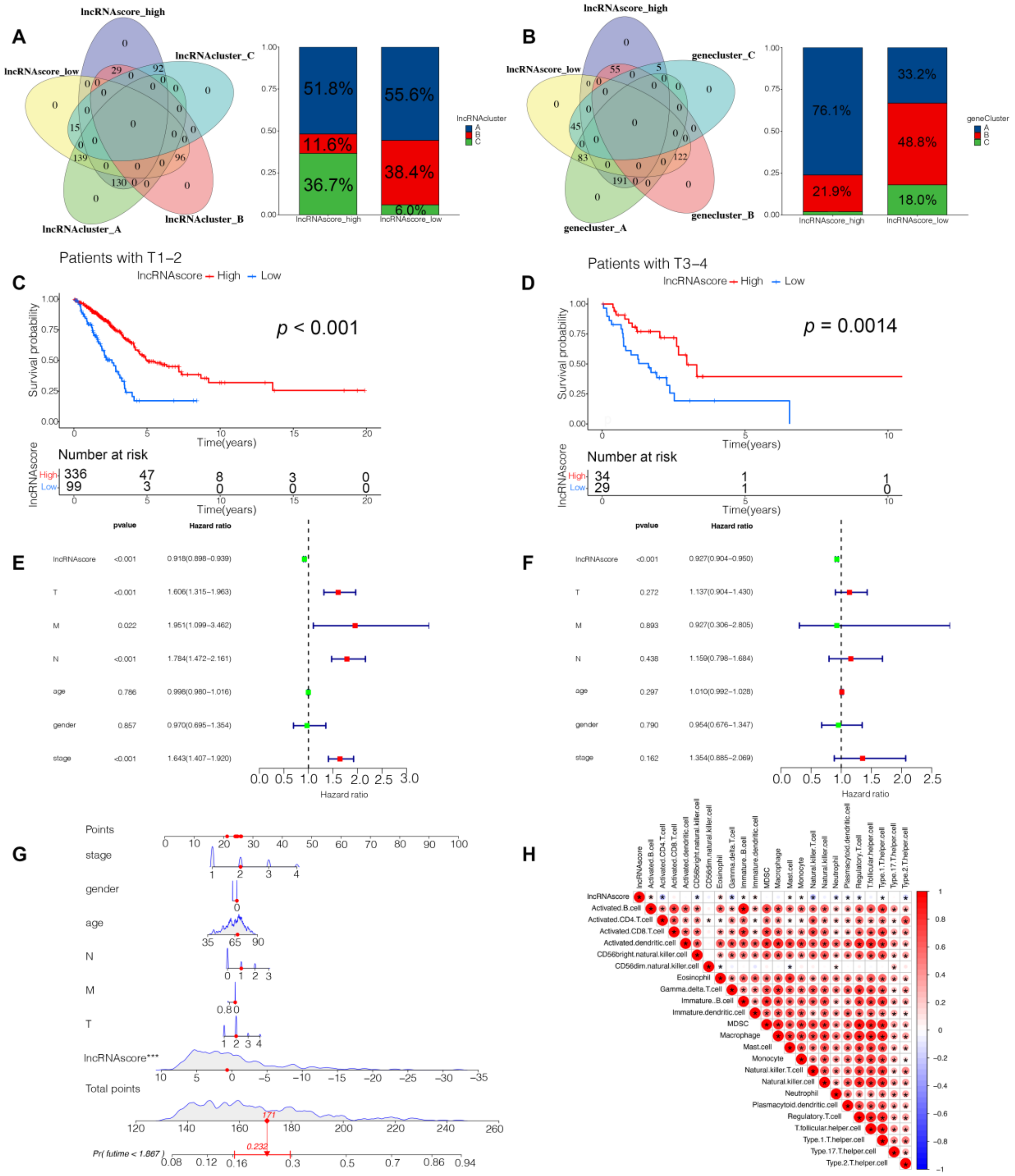

Figure S4. Prognostic value of lncRNA score and correlation between the clinicopathological features and lncRNA score.
